# Dissolved organic carbon in glaciers of the southeastern Tibetan Plateau: Insights into concentrations and possible sources

**DOI:** 10.1371/journal.pone.0205414

**Published:** 2018-10-11

**Authors:** Yulan Zhang, Shichang Kang, Gang Li, Tanguang Gao, Pengfei Chen, Xiaofei Li, Yajun Liu, Zhaofu Hu, Shiwei Sun, Junming Guo, Kun Wang, Xintong Chen, Mika Sillanpää

**Affiliations:** 1 State Key Laboratory of Cryospheric Science, Northwest Institute of Eco-Environment and Resources, Chinese Academy of Science, Lanzhou, China; 2 Laboratory of Green Chemistry, Lappeenranta University of Technology, Mikkeli, Finland; 3 University of Chinese Academy of Science, Beijing, China; 4 CAS Center for Excellence in Tibetan Plateau Earth Sciences, Beijing, China; 5 Arid Meteorological Research Institute, Lanzhou Meteorological Bureau, Lanzhou, China; 6 Key Laboratory of Western China's Environmental Systems (Ministry of Education), College of Earth and Environmental Sciences, Lanzhou University, Lanzhou; Universita degli Studi di Milano, ITALY

## Abstract

Dissolved organic carbon (DOC) released from glaciers has an important role in the biogeochemistry of glacial ecosystems. This study focuses on DOC from glaciers of the southeastern Tibetan Plateau, where glaciers are experiencing rapid shrinkage. We found that concentrations of DOC in snowpits (0.16±0.054 μg g^−1^), aged snow (0.16±0.048 μg g^−1^), and bare ice (0.18±0.082 μg g^−1^) were similar across the southeastern Tibetan Plateau, but were slightly lower than those in other glaciers on the Tibetan Plateau. Vertical variations of DOC, particulate organic carbon, black carbon, and total nitrate in snowpit showed no systematic variations in the studied glaciers, with high values of DOC occurring in the ice or dusty layers. We estimated the export of DOC and particulate organic carbon from glaciers to be 1.96±0.66 Gg yr^–1^ and 5.88±2.15 Gg yr^–1^ in this region, respectively, indicating that organic carbon released from glacier meltwater may be affecting downstream ecosystems. Potential sources of the air masses arriving at the southeastern Tibetan glaciers include South Asia, Central Asia, Middle East, and northwest China. Emissions from biomass burning of South Asia played an important role in the deposition of DOC to the glacier, which can be evidenced by backward trajectories and fire spot distributions from MODIS and CALIPSO images. Our findings suggest that anthropogenic aerosols contribute abundant DOC to glaciers on the southeastern Tibetan Plateau. The pronounced rate of glacial melting in the region may be delivering increased quantities of relic DOC to downstream rivers.

## Introduction

Glaciers cover a significant portion of the Earth’s surface and form an integral part of the global climate system [1–3]. Because of the various inorganic and organic compounds deposited on them and the physical and photochemical processes that occur within them, glaciers can serve as a reservoir of atmospheric chemistry [4], and their retreat can affect carbon cycle and water resources [5–6]. Relatively few studies focus on organic carbon (OC) in glaciers as compared to studies on inorganic components (i.e., major ions, elements) [7], thus OC is one of the least understood fractions in snow and ice [1].

Because it makes up a large portion of the OC in glaciers [1, 8], dissolved organic carbon (DOC) plays a fundamental role in the biogeochemistry of the glacier system [9]. Hood et al. [1] estimated that the DOC stored in glaciers was about 4.48±2.79 Pg C, constituting about 75% of the OC stored in glaciers and ice sheets. The concentrations and quality of DOC in glaciers varies considerably, with high variability both temporally across the glacier meltwater season [10–11], and spatially across different regions [8, 12–13]. Hood et al. (2015)[1] found that DOC concentrations on mountain glaciers, and on the Greenland and Antarctic ice sheet ranged from 0.01 to 43.2 μg g^−1^ with a mean of 0.97 μg g^−1^. This highlights glaciers as ecosystem heavily depleted in OC [14].

Glaciers accumulate organic OC from in situ primary production as well as from atmospheric deposition of carbonaceous material derived from terrestrial and anthropogenic inputs [1, 15]. Anthropogenic combustion products are considered the main source of the aerosol organic carbon deposited on glacier surfaces [9–10, 16]. Other possible sources of OC in glacier include aerosols from forest fires [10, 17–18], soil organic matter (Singer et al., 2012; Yan et al., 2016), and biological processes [19–21]. This diversity of sources was documented by Antony et al. [19] and Fellman et al. [9] in recent molecular level analyses of DOC in snow and its dual-carbon-isotope signatures (δ^13^C/Δ^14^C).

Hood et al. [20] and Singer et al. [14] found that glacier-derived DOC represents a quantitatively significant source of bioavailable carbon to downstream carbon cycling in glacier-fed streams. The Greenland ice sheet exports labile OC (0.13−0.17 Tg C yr^−1^) to the Arctic Ocean and may represent an important OC source to the near-coastal North Atlantic, Greenland, and Labrador seas [22]. Molecular insights on DOC transformation by supraglacial microbial communities have indicated that both autochthonous and allochthonous dissolved organic matter is highly bioavailable and is transformed by resident microbial communities through parallel processes of degradation and synthesis [15].

The Tibetan Plateau contains a large volume of glaciers, and plays a significant role in the Earth’s climate system [5, 23]. The atmospheric circulation patterns over the Tibetan Plateau are characterized by the South Asian (Indian) monsoon in summer and the westerlies in winter [24]. The atmospheric circulation patterns over the Tibetan Plateau are characterized by the South Asian (Indian) monsoon in summer and the westerlies in winter [24]. Currently, glaciers in the Tibetan Plateau are experiencing rapid retreat [5, 23], particularly in the southeastern Tibetan Plateau [5]. Temperate glaciers are typical in the southeastern Tibetan Plateau and account for approximately 20% of the total number of glaciers in China [25]. Because they have exhibited strong surface melting and rapid terminal retreating, these glaciers are subject to extensive mass loss [5]. The water released by glaciers in this region is important to the downstream countries [26]. For instance, glacial meltwater can contribute more than 50% of the total runoff increase in the upper Brahmaputra [27]. In the Indus and Ganges basins, about 40% of the meltwater originates from glaciers [28]. Kääb et al. [29] and Gardner et al. [30] estimated that glaciers in the Himalayan region lost 24±2 Gt yr^−1^ of ice between 2003 and 2009, equivalent to around 10% of the global glacier mass. Climate-driven changes in glacier volume will alter downstream discharge, the speciation of nutrients, and societal development [2, 26]. Previous studies of DOC in Tibetan glaciers mainly focused on samples from snow pits [13, 31–33]. In this study, snow pit and surface snow/ice samples were collected from glaciers in the southeastern Tibetan Plateau and analyzed for DOC, particulate organic carbon (POC), total nitrate (TN), black carbon (BC), major ions, and elements. We will describe the characteristics of DOC and POC from glaciers on the southeastern Tibetan Plateau, and assess DOC and POC deposition on glaciers and contribution to river runoff. Finally, we also discuss possible sources of DOC in the southeastern Tibetan Plateau. Resolving the characteristics and origin of DOC on the southeastern Tibetan Plateau is important for understanding how the storage and release of OC from glaciers will change in the future. These results will benefit local people and attract public attentions to the cryospheric change in the Tibetan Plateau.

## Methodology

### Field site and snow sampling

**Fig 1:**
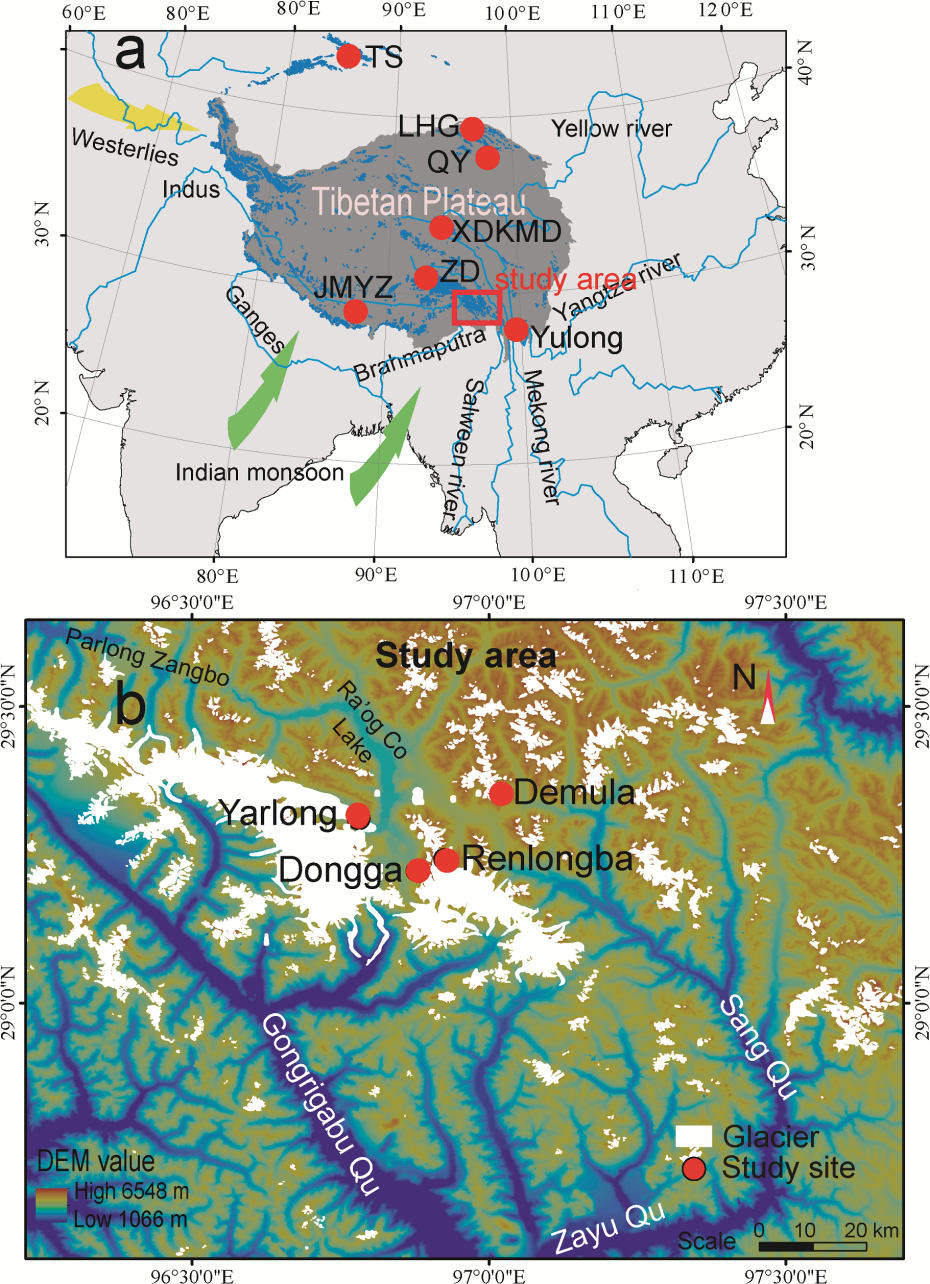
(a) Location of the study area and (b) the distributions of studied glaciers in the southeastern Tibetan Plateau. (Red dots in (a) represent the glaciers referenced in the main text. TS: Urumqi Glacier No. 1 in Tienshan; LHG: Laohugou No.12 glacier in Qilian Mountains; QY: Qiyi glacier in Qilian Mountains; XDKMD: Xiaodongkemadi glacier in the Tanggula Mountains, central Tibetan Plateau; ZD: Zhadang glacier in the Mt. Nyainqengtanlha; JMYZ: Jiemayangzong glacier in the Himalayas; Yulong; Yulong snow mountain in the southeastern Tibetan Plateau).

This study is under the permit of Northwest Institute of Eco-Environmental and Engineering Research, Chinese Academy of Sciences. Field sampling sites in our study did not involve endangered or protected species. The results will benefit local people and attract public attentions to the cryospheric change in the Tibetan Plateau. The study area is located along the southeastern margin of the Tibetan Plateau (Fig 1A). This region is strongly influenced by the South Asian monsoon, which typically arrives via the Brahmaputra Valley [34]. During expeditions in June of 2015, fresh snow, aged snow, and bare ice samples (the upper 4−8 cm) were collected from four glaciers (Yarlong (29°18ʹ20.23ʺN, 96°46ʹ26.88ʺE, 4050 m a.s.l.), Dongga (29°13ʹ34.97ʺN, 96°52ʹ30.53ʺE, 4700 m a.s.l.), Renlongba (29°14ʹ33.51ʺN, 96°55ʹ29.67ʺE, 4850 m a.s.l.), and Demula (29°21ʹ09.33ʺN, 97°01ʹ14.22ʺE, 5140 m a.s.l.)) with average elevations ranging from 4030 to nearly 5090 m a.s.l. (Fig 1B and S1 Table). In addition, three snowpits were collected from the Dongga (120 cm), Renlongba (70 cm), and Demula (80 cm) glaciers (detailed in Dataset). Among them, snowpits of Renlongba and Demula glacier were excavated at a vertical depth of 10 cm; while snowpit of Dongga glacier was excavated at a vertical depth of 15 cm using a stainless steel spoon. Generally, in order to eliminate contamination, polycarbonate vials (polycarbonate) were firstly washed using ultrapure water, soaking with 1 M HCl overnight, rinsing using ultrapure water again, and finally soaking in ultrapure water for 24 h [33, 35]. Surface snow and snowpit samples were directly collected into pre-cleaned polycarbonate vials for DOC analysis. Polyethylene plastic bags were used to collect snow samples (approximately 2 L, unmelted) for analysis of BC, POC, a major ions, and elements [36]. During sampling, dust-proof garment with a head-and-mouth mask and gloves were worn to assure samples were not contaminated [37]. In total, we collected 18 surface snow/ice samples and 23 snow pit samples, and used parallel samples to average the results. Laigu river water samples for DOC analysis were collected from 20:00, 19 June to 18:00, 20 Jun 2015, every 2 h in the Yarlong glacier region (Fig 1B). River water was directly collected into pre-cleaned polycarbonate vials. Field these sampling sites in our study did not involve endangered or protected species.

All samples were kept frozen until analysis. Before filtration, the samples in plastic bags were rapidly melted in a water bath (approximately 20 minutes for complete melting) and the melt water (typically 1 L) was filtered through a pre-dried (in a desiccator, at 550°C, for 6 hours), weighted quartz substrate using a vacuum pump. We filtered the samples twice, rinsing the filtration equipment twice with ultrapure water (<18.2 mΩ) to avoid particle loss. Finally, we transferred the filtered water into pre-cleaned polyethylene vials for the analyses of major ions and elements and used the filters for POC and BC analyses [36].

### Analytical protocols

#### DOC measurement

We determined DOC concentrations of the glacial samples by high-temperature combustion (680°C) using a Total Organic Carbon analyzer (TOC-5000A, Shimadzu Corp, Kyoto, Japan) [16, 33] after filtration through a polytetrafluoroethylene (PTPE) membrane filter (0.45 μm pore size). For liquid water samples, the detection limit of the analyzer was 15 μg L^–1^ with a standard deviation less than 1.5%. Average DOC and TN of blanks was 33±10 and 28±6 μg L^–1^, respectively, indicating that contamination during sampling, pre-treatment, and analysis processing could be ignored.

#### POC and BC analysis

After filtering, we analyzed the quartz filters for POC and BC. Based on an adapted IMPROVE protocol [38–39], we measured the POC and BC on the filters using a thermal-optical carbon analyzer. Because the dust loads in the snow/ice samples were greater than in the airborne aerosol samples, we modified the method such that, in a 100% helium atmosphere, only one temperature plateau (550°C) was used to reduce the time that the BC was exposed to the catalyzing atmosphere [36, 39]. The detection limit of the analysis was 0.19±0.13 μg total carbon (TC) cm^–2^ and the filter blank was 1.23±0.38 μg TC cm^–2^, which was much lower (less than one order of magnitude) than the measured sample values.

#### Major ions and elements

We measured major ions (Na^+^, K^+^, Ca^2+^, Mg^2+^, NH_4_^+^, Cl^−^, SO_4_^2−^, NO_3_^−^) using a Dionex-600 and Dionex-2500 Ion Chromatograph System (Dionex, USA). The detection limit was 1 μg L^–1^, and the precision was less than 5%.

In the laboratory, we measured elements directly using inductively coupled plasma-mass spectrometry (ICP-MS, X-7 Thermo Elemental). The snow samples were melt in the laboratory at room temperature, and then acidified with 1% HNO_3_ and allowed them to react with the acid for seven days before being measured. We quantified elemental concentrations using external calibration standards (AccuTrace Reference Standard), analyzing the analytical standard after the initial calibration and after every 10 samples. We defined the method detection limit (MDL) as three times the standard deviation of replicate blank measurements. The MDL for the elements are given in the supplementary material published by Zhang et al. [40]. Detailed information about the elemental analysis can also be found in Cong et al. [41].

### Backward air mass trajectories

We calculated backward air mass trajectories using the Hybrid Single-Particle Lagrangian Integrated Trajectory (HYSPLIT) model (Version 4) [42]. We use 5-day-long backward trajectory with a daily resolution to simulate the moving routes of air masses arriving at the sampling sites (ending at 12:00 (04:00 UTC) Beijing time for the sampling period). The backward trajectories were calculated at 1000 m above ground level. The trajectory frequency option started a trajectory from a single location and height every 6 hours, summed the frequency that the trajectory passed over a grid cell, and normalized it by using either the total number of trajectories or endpoints. A trajectory may intersect a grid cell once or multiple times.

### Principal component analysis

Principal component analysis (PCA) is a statistical procedure that uses an orthogonal transformation to convert a set of observations of possibly correlated variables into a set of values of linearly uncorrelated variables called principal components. A biplot is also drawn, which is regarded as a graphical display of matrix multiplication [43].

### CALIPSO images

Cloud-Aerosol Lidar and Infrared Pathfinder Satellite Observations (CALIPSO) data can provide vertical distributions of aerosol and cloud profiles in Earth’s climate system [44]. Previous studies indicated that CALIPSO images can capture the features of atmospheric pollutants transportation [45].

## Results

### Abundance of DOC and other chemicals

We found average concentrations of DOC of 0.16±0.05, 0.16±0.05, and 0.18±0.08 μg g^−1^ for snow pit, aged snow, and bare ice samples, respectively (Table 1). No significant variations were exhibited between different snow types. The relatively high DOC concentrations for bare ice may be a result of increased impurities in these samples. Compared with the other studies noted in Table 1, DOC concentrations were somewhat lower than those from elsewhere on the Tibetan Plateau, comparable to those from surface snow in the French Alps and Alaska, and higher than those from snow of the Greenland ice sheet. The average POC concentrations in bare ice (0.71±0.82 μg g^−1^) of the glaciers we studied were higher than those from snow pit and aged snow samples and much higher than those from ice core records (Table 2). Snow pit POC data were comparable to those from margin areas of the Tibetan Plateau (Yulong and Laohugou No.12 glacier), but higher than those from the central Tibetan Plateau (Zhadang and Xiaodongkemadi glacier). The POC concentrations in aged snow (0.38±0.60 μg g^−1^) were lower than those from Yulong Snow Mountain, Zhadang glacier, Xiaodongkemadi glacier, Laohugou No.12 glacier, and Keqikaer glacier (Table 2). We found no significant relationships between DOC (or POC) concentrations and elevations; however, the DOC and POC concentrations in the glacier surface snow and ice may be influenced by other complex factors, such as slope [46], cryoconite holes [47–48], or biological activities [47, 49]. The DOC concentrations we observed were within the range of previously reported values for the glacierized regions outside the Tibetan Plateau [1].

**Table 1:** Comparison of DOC concentrations from glaciers in the Tibetan Plateau and other remote areas.

Region	Year	Snow types	DOC conc. (μg g^−1^)	Comment	References
Southeast Tibetan Plateau	Jun, 2015	Snowpit	0.16±0.054	Shimadzu TOC-5000A Total Organic Carbon analyzer	This study
		Aged snow Bare ice	0.16±0.048 0.18±0.082		
Musidao glacier, Altai	Aug, 2014	Snowpit	0.76±0.19	Vario EL CN analyzer	[31]
Urumqi No.1 glacier, Tienshan	Aug, 2014	Snowpit	0.52±0.14	Vario EL CN analyzer	[31]
LHG, Northern Tibetan Plateau	Jul-Aug, 2015	Snowwpit	0.33±0.13	Shimadzu TOC-5000A Total Organic Carbon analyzer	[31]
LHG, Northern Tibetan Plateau	Aug, 2016	Fresh snow Snowpit	0.38±0.06 0.22±0.11	Shimadzu TOC-5000A Total Organic Carbon analyzer	[50]
LHG, Northern Tibetan Plateau	Aug, 2014	Snowpit	0.66±0.08	Vario EL CN analyzer	[31]
Qiyi, Northern Tibetan Plateau	Jun, 2014	Snowpit	0.65±0.52	Shimadzu TOC-5000A Total Organic Carbon analyzer	[13]
XDKMD, Central Tibetan Plateau	Aug, 2014	Snowpit	0.91±0.20	Vario EL CN analyzer	[31]
XDKMD, Central Tibetan Plateau	Jun, 2014	Snowpit	0.59±0.32	Shimadzu TOC-5000A Total Organic Carbon analyzer	[13]
Zhadang, Southern Tibetan Plateau	Aug, 2014	Snowpit	1.26±0.09	Vario EL CN analyzer	[31]
Yulong, Southern Tibetan Plateau	Jun, 2014	Snowpit	0.48±0.05	Shimadzu TOC-5000A Total Organic Carbon analyzer	[13]
Yulong, Southern Tibetan Plateau	Jun, 2015	Snowpit Aged snow	0.54±0.22 0.90±0.16	Vario TOC select, Germany	[51]
JMYZ, Southern Tibetan Plateau	Summer 2009 Winter-Spring	Snowpit Snowpit	0.61 2.68	Shimadzu TOC-VCPH	[32]
Mt. Blanc, French Alps	Sep, 2012	Surface snow	0.21±0.01	UV/NDIR CO_2_	[8]
Juneau Icefield, Southeast Alaska	May, 2013	Glacier surface snow	0.20	Shimadzu TOC-V CSH analyzer	[9]
Mendenhall Glacier, Alaska		Snowpit	0.19	OI Analytical 700 TOC analyzer	[16]
Bow lake, Albert, Canada		Glacier stream	0.35±0.15	Shimadzu TOC 5000A analyzer	[49]
Greenland Ice Sheet	May-Aug, 2012	Snow Ice	0.06 0.18	Shimadzu TOC-VCSN/TNM-1 Analyzer	[50]

**Table 2:** POC concentrations from different glaciers in the Tibetan Plateau and its surroundings.

Region	Year	Snow types	POC conc. (μg g^−1^)	Comment	References
Southeast Tibetan Plateau	Jun, 2015	Snowpit	0.41±0.52	TOR method, DRI2001A	This study
		Aged snow Bare ice	0.38±0.60 0.71±0.82		
Zuoqiupu, Southeast Tibetan Plateau	1960–2005	Ice core	0.01–0.04	TOR method, DRI2001A	[52]
Yulong, Southeast Tibetan Plateau	Jun, 2015	Snowpit Aged snow	0.57±0.10 2.09±0.57	TOR method, DRI2001A	[51]
Zhadang, South Tibetan Plateau	Aug, 2015 May, 2015	Fresh snow Aged snow	0.14±0.0.02 1.38±0.37	TOR method, DRI2001A	[13]
XDKMAD, Central TP	Aug 2014 to Oct 2015	Fresh snow Aged snow	0.16±0.04 0.61±0.47	TOR method, DRI2001A	[53]
LHG, Northern TP	Jun, 2016	Snow pit Aged snow	0.39±0.22 0.53±0.29	TOR method, DRI2001A	[54]
KQKE, Tienshan	May, 2015	Aged snow	1.74	TOR method, DRI2001A	[55]

**Fig 2:**
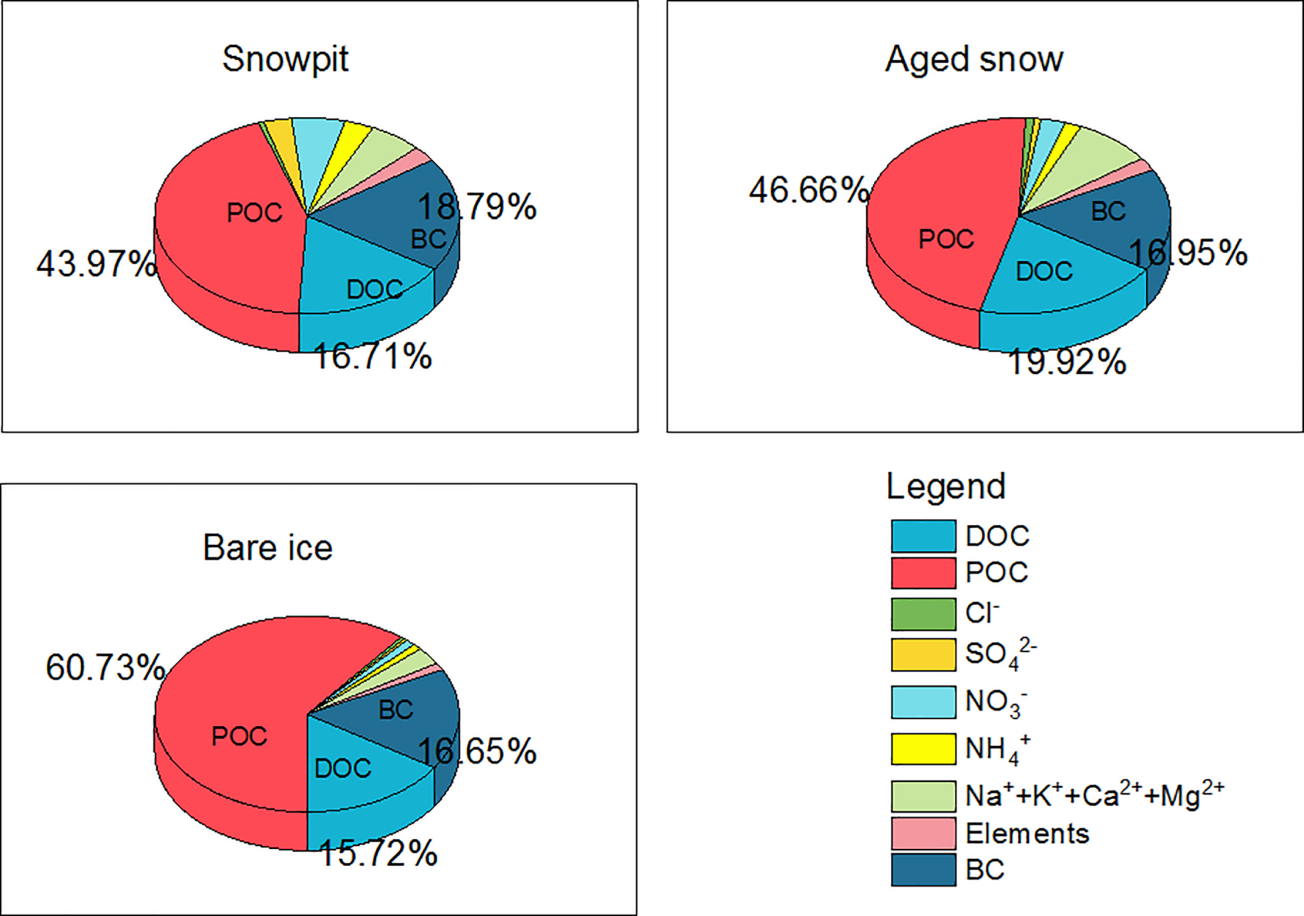
Average component proportions in snow pit, aged snow, and bare ice, southeastern Tibetan glaciers.

The mass fraction of organic and inorganic components in snow we measured indicated similar proportions of DOC for snow pit, aged snow, and bare ice (Fig 2), implying DOC levels in snow were not controlled only by snowmelt. The highest percentage of POC (> 60%) was found in bare ice, but the highest percentage of BC occurred in snow pits, indicating differing post-deposition processes. Carbonaceous components dominated the impurities in snow, contributing more than 80% of mass fractions. The much higher portion of DOC in snow may indicate different sources or post-deposition processes.

**Fig 3:**
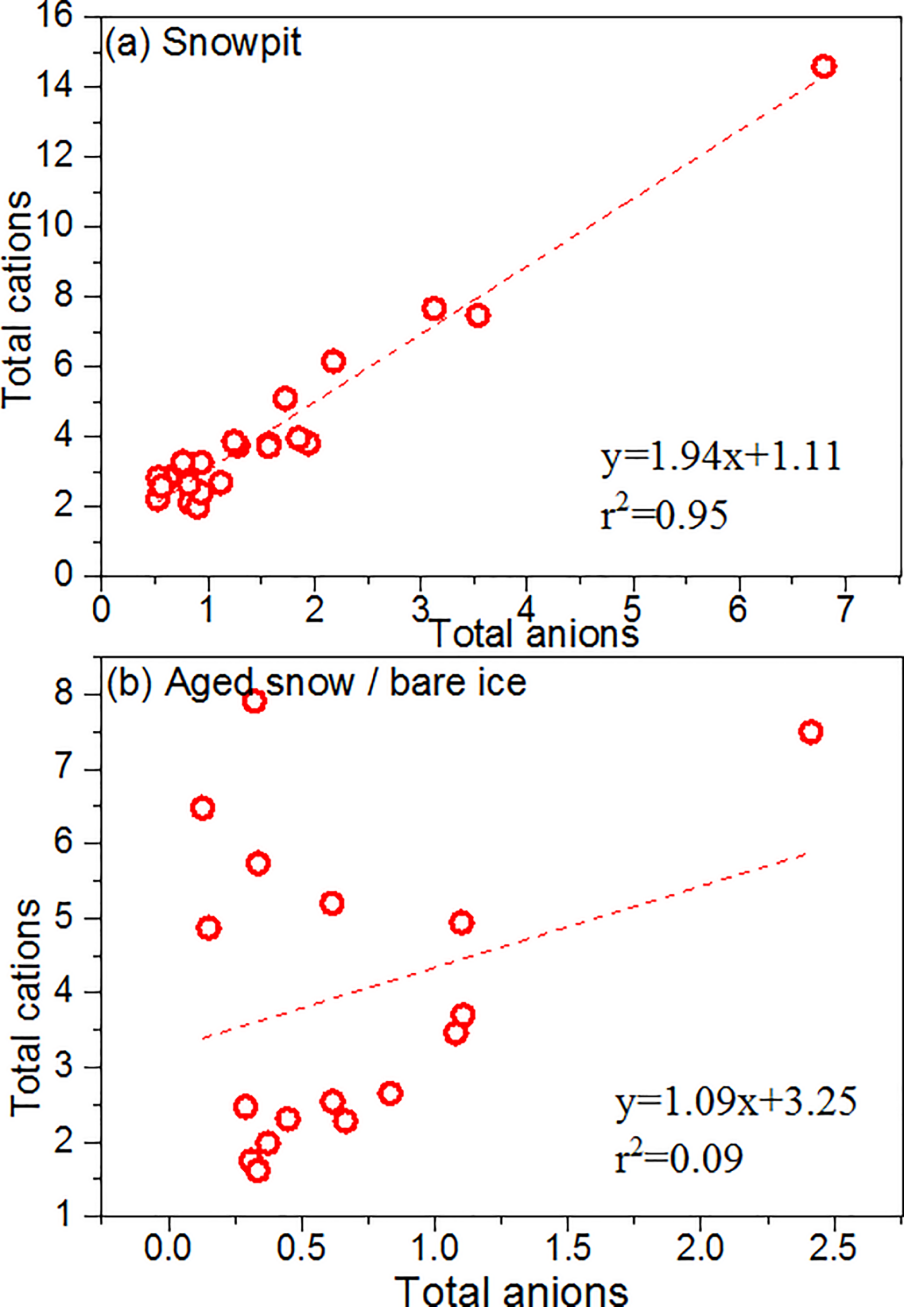
Correlations between total cations (∑+) and total anions (∑–) in (a) snow pit and (b) aged snow/bare ice samples.

Water soluble major ions accounted for 17.95%, 14.28%, and 5.67% of the total mass for snow pit, aged snow, and bare ice, respectively (Fig 2). The portion of [NO_3_^−^+NH_4_^+^] and [Na^+^+K^+^+Ca^2+^+Mg^2+^] were highest in the snow pit samples and lowest in the bare ice samples, indicating that glacier melt water eluviation can remove most major ions from snow. The charge balance between the total cations (∑+) and total anions (∑–) is shown in Fig 3. Strong correlations between the two totals for snow pit samples indicate most of the ionic components were measured, and the fact that the average ∑+/∑–ratio was larger than unity implies that the glacier snow water was slightly alkaline, as was also found by Zhang et al. [7]. For aged snow and bare ice samples, the correlation between ∑+ and ∑–was not significant and may have been affected by the glacier meltwater elution or post-deposition processes.

### Vertical variations

**Fig 4:**
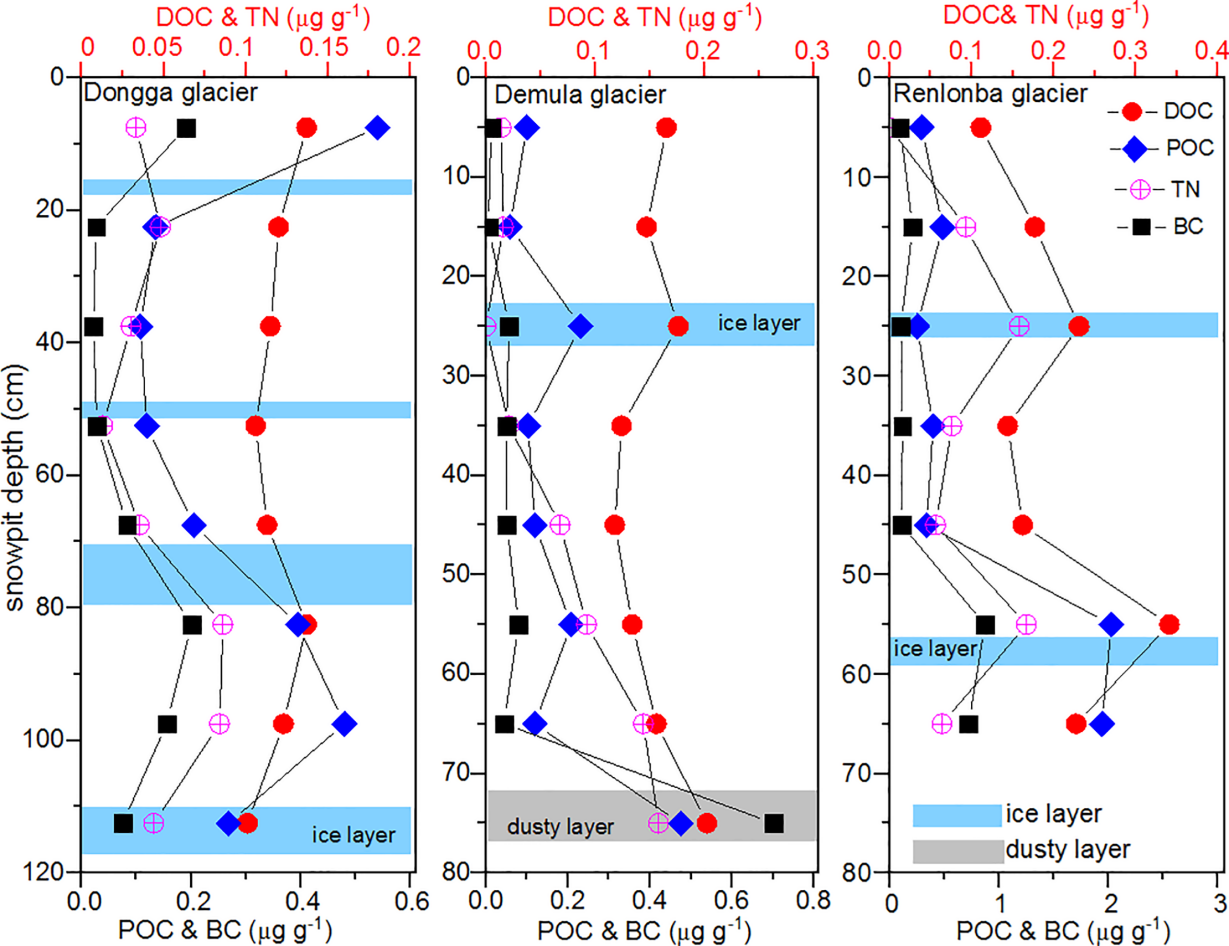
Vertical variations of DOC, POC, BC, and TN from snow pits collected from southeastern Tibetan Plateau.

We found no systematic variations for DOC and TN with snow pit depth in the studied glaciers, but POC and BC showed consistent variations (Fig 4). High DOC values occurred in the ice layer or dusty layers, similar to the patterns observed by Yan et al. [33] on the Laohugou No. 12 glacier in the northern Tibetan Plateau and by Xu et al. [32] on the Jiamayangzong glacier in the southern Tibetan Plateau. This indicates that DOC concentrations in the study area were likely influenced by mineral dust deposition. Doherty et al. [56] noted that BC-containing particles were washed through the snow pit with higher efficiency than larger particles, such as soil or mineral dust. Benning et al. [57], Takeuchi et al. [58], and Yang et al. [39] noted that BC and POC redistribute vertically in glaciers, potentially under the control of melting processes or biological melt enhancement effects.

### DOC and POC export from glaciers

The amount of DOC stored in glaciers can be determined from their mass of water and average DOC concentrations (DOC_avg_) [1]. In the southeastern Tibetan Plateau, we estimated the export of DOC and POC from glaciers using Eq (1):

$$Export\;DOC={DOC}_{avg}\times MB\times GA$$ (1)

Where, MB represents the annual mass balance (mm w.e. yr^-1^), and GA means the glacier area (km^2^). Yao et al. [5] estimated the annual mass balance in the southeastern Tibetan Plateau to be 1100 mm w.e. and estimated the glacier area from the Hengduan and Nyainqengtanglha mountains at 10,699 km^2^. Based on Eq (1) and Table 1, we estimated the export of DOC is 1.96±0.66 Gg yr^–1^ in the study areas (or southeastern Tibetan Plateau). The export of POC from the study area were calculated to be 5.88±2.15 Gg yr^–1^, much higher than DOC export due to higher POC concentrations from glaciers.

## Discussions

### Potential sources of DOC

**Fig 5:**
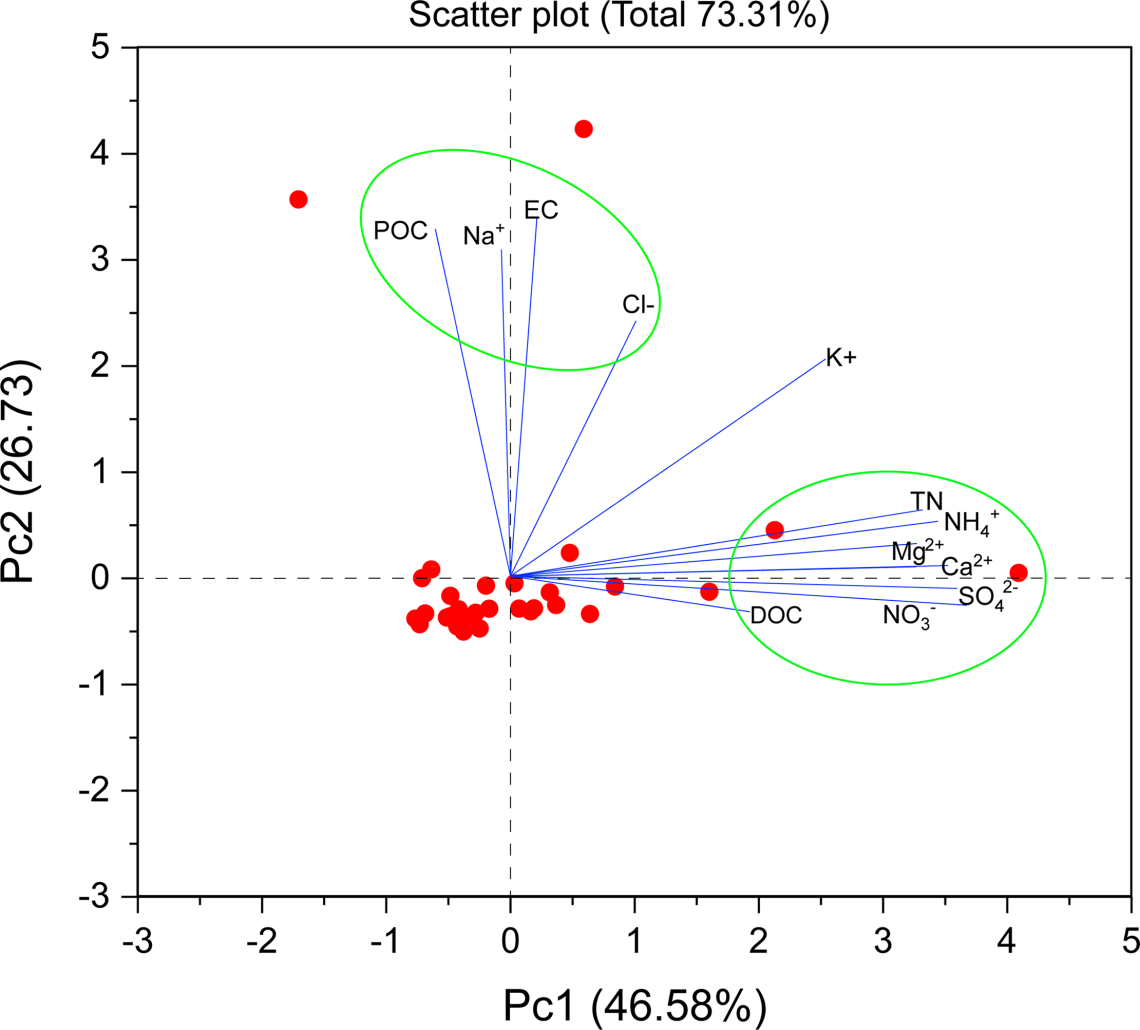
Biplot to show similarities among measured glacial proxies in discriminating the relationships.

The results of PCA for DOC and other proxies for glaciers of the southeastern Tibetan Plateau showed that four principal components differed significantly (see S2 Table), reflecting complex associations between different chemicals in glaciers. The first PCA (PCA1), mainly loaded by POC, EC, Na^+^, K^+^, and Cl^–^, accounted for 46.58% of total variance and may reflect the relatively consistent aerosol deposited on the glaciers. PCA2, loaded by Ca^2+^, Mg^2+^, and SO_4_^2–^ (accounting for 26.73% of total variance), indicated crustal sources from desert or arid regions as noted by Zhang et al. [7]. TN, NO_3_^–^, and NH_4_^+^ were included in PCA3 (contributed 12.34% to total variance), indicating the impact of anthropogenic activities. Unlike other parameters in glaciers, DOC was the primary component in PCA4 with an extraction of 62.41%, and was loaded with Cl^-^ and K^+^.Biplot of PAC explained 73.31% of total variation of environmental proxies in this study (Fig 5). As shown in Fig 5, the cosine of the angle between the two environment vectors (red lines) approximates the correlation between them. For example, Ca^2+^, Mg^2+^, and SO_4_^2-^ were positively correlated with each other (acute angle); DOC is also positively related to with Ca^2+^, Mg^2+^, and SO_4_^2-^, indicating impact of crustal sources. The positive relationship between DOC and K^+^ (acute angle) indicated impact of biomass burning [59]. Deposition of Cl is a proxy for sea-salt deposition, implying impact of sea-salt aerosol on DOC (acute angle) deposition in the southeastern Tibetan Plateau. The distance between the proxies measures their dissimilarity. Thus, the 12 proxies fell into two groups (green circles in Fig 5).

**Fig 6:**
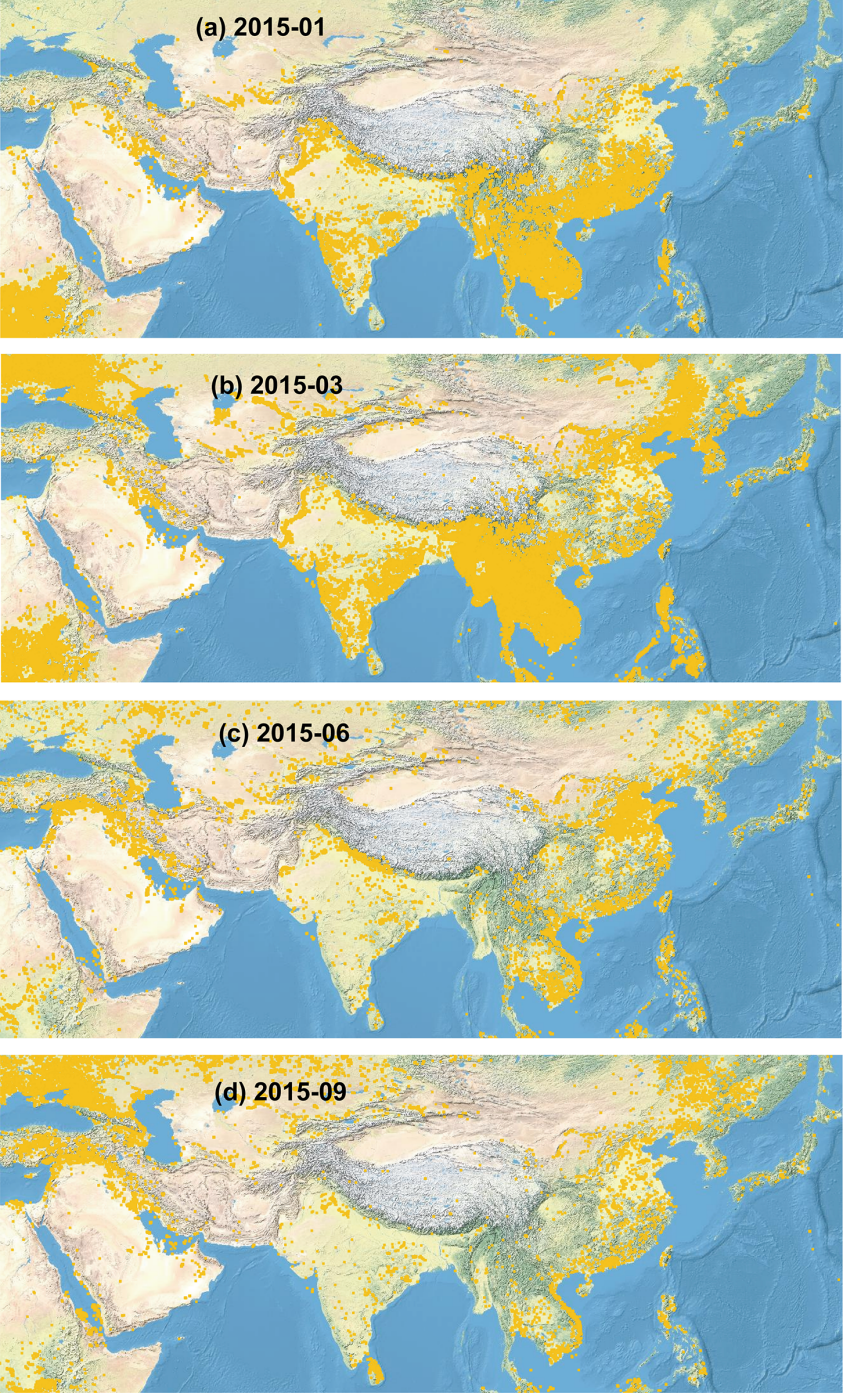
Fire spot distribution from December 2014 to September 2015 as determined by MODIS (moderate resolution imaging spectroradiometer) (https://firms.modaps.eosdis.nasa.gov/map/).

**Fig 7:**
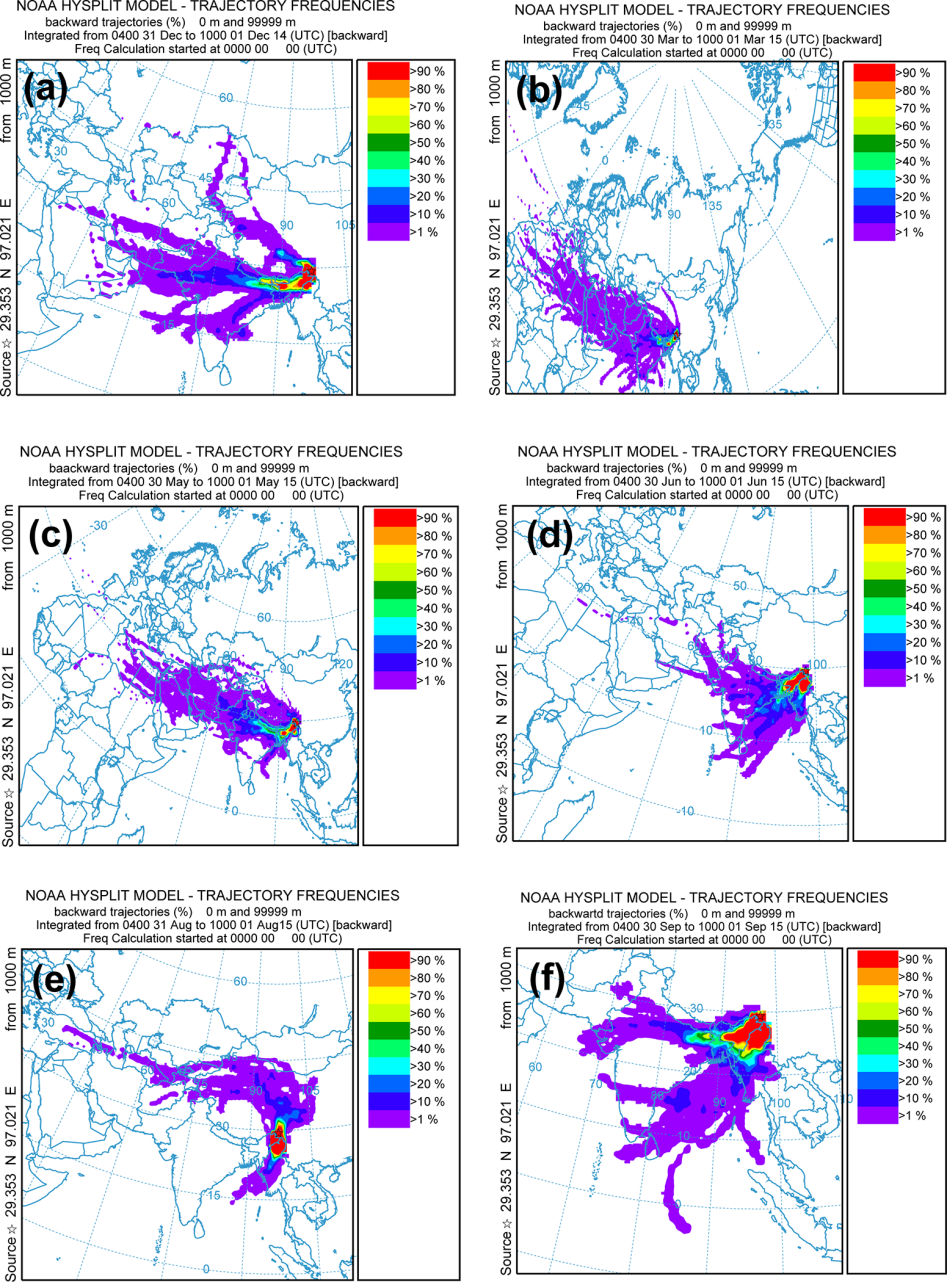
Distributions of backward trajectory frequency from December 2014 to September 2015. (https://ready.arl.noaa.gov/HYSPLIT.php).

The southeastern Tibetan Plateau is adjacent to South and Southeast Asia (Fig 6), which experience widespread biomass burning (e.g., forest fire, straw burning) [60–61]. Biomass burning is more frequent and widespread in winter and pre-monsoon seasons (Fig 6A and 6B) than in summer and post-monsoon season (Fig 6C and 6D). Biomass burning is considered to be an important source of aerosols transported to the Himalayas and Tibetan Plateau [59, 62]. Backward trajectory frequency shows that during the winter and pre-monsoon seasons, air masses arriving at the southeastern Tibetan Plateau originate primarily from South Asia (including the Thar Desert region), Central Asia, the Middle East, and the western Tibetan Plateau (Fig 7A, 7B and 7C). In June, air masses originate mainly from South Asia and the Bay of Bengal (Fig 7D). Our analyses showed that during summer, air masses from the northern Tibetan Plateau and northwest China play an important role in the aerosol deposition on the glaciers of the southeastern Tibetan Plateau (Fig 7E). In September (post-monsoon season), air masses are again generally sourced from South Asia and the Bay of Bengal (Fig 7F). When biomass burning occurs in the air mass source regions (e.g., South Asia), the aerosol can be transported and deposited on the Tibetan glacier surface. This indicates that forest fires [10, 17–18] and soil organic matter [14, 33] are important sources of OC found in glaciers. In the central Himalayas, Cong et al. [59] observed strong positive correlations for dicarboxylic acids with biomass burning tracers, levoglucosan and K^+^, demonstrating that this area can be affected by biomass burning from South Asia. In addition, local contribution (e.g., biofuel burning of local people) may also contributed to OC deposition in glaciers of this regions.

**Fig 8:**
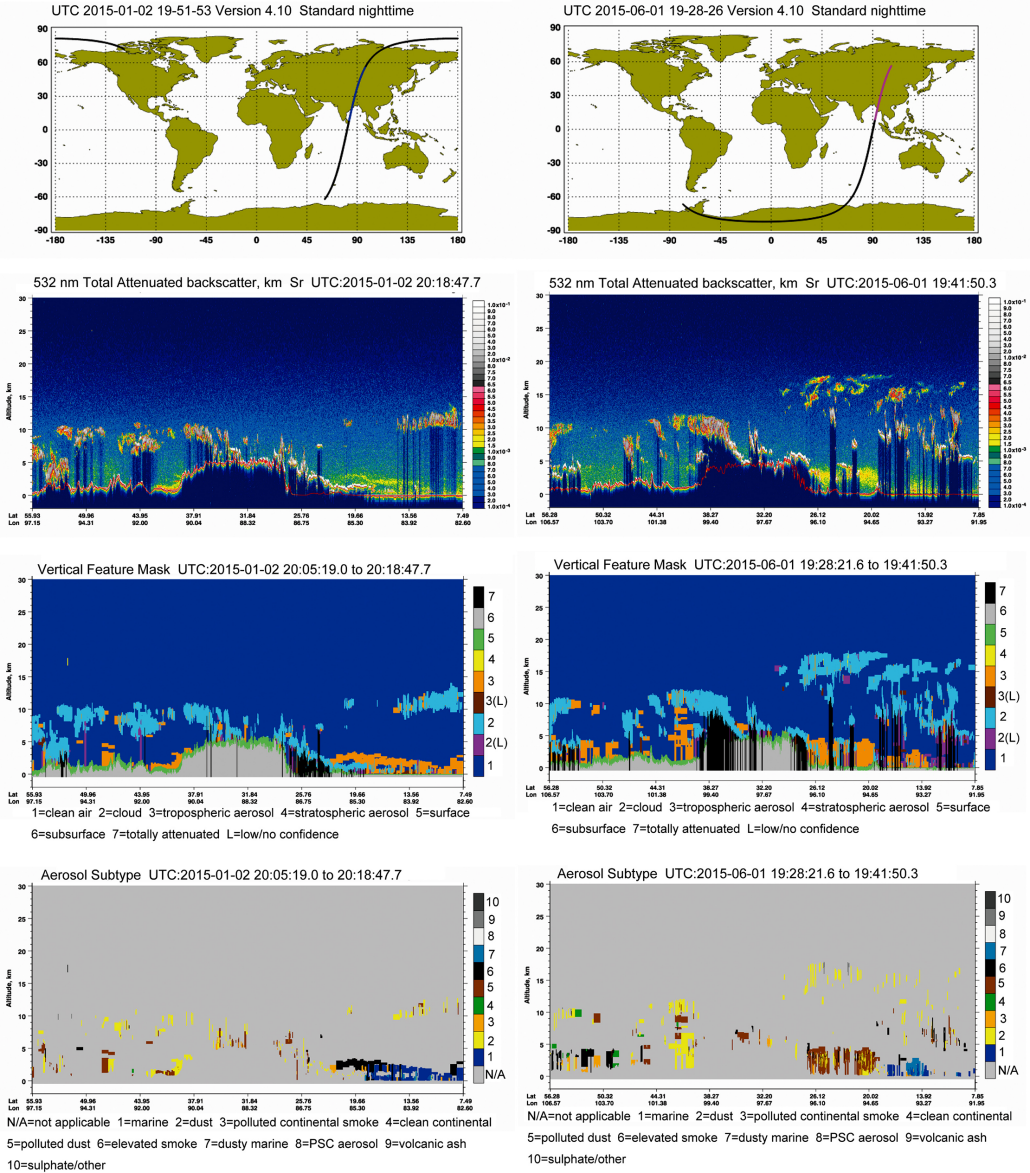
CALIPSO-retrieved backscatter signal at 532 nm, vertical feature mask, and aerosol sub-type information on 2 January 2015 and 1 June 2015. The southeastern Tibetan Plateau (marked with circles) is covered by a thick aerosol layer, suggesting that air pollutants could extend more than 5 km in altitude. CALIPSO profiles were obtained from (https://www-calipso.larc.nasa.gov/).

CALIPSO-derived 532 nm backscatter values for our study area, as well as smoke plume heights and aerosol sub-types with plume heights, are shown in Fig 8. Vertical profiles of aerosol concentrations reveal large values over South Asia. Aerosol subtypes demonstrate that smoke plumes could extend higher than 5 km in altitude. An example of such pollution phenomenon was observed on 2 January 2015 and 1 June 2015 (Fig 8), clearly demonstrating that the southeastern Tibetan Plateau (marked with circles) is covered by a thick polluted aerosol layer, apparently originating from South Asia.

### Estimation of DOC and POC export

In a previous study, Liu et al. [31] estimated that the total amount of DOC stored over the Tibetan Plateau is ∼3.96 ± 0.87 Tg, approximately ~5.6% of the total DOC in alpine glaciers (70 Tg) around the world [1]. Pfeffer et al. [63] found DOC released from glaciers in the high mountains of Asia to be 30.8 Gg yr^–1^, 50% of which was contributed by glaciers in China. Because different DOC concentrations and area were used, our estimation of DOC export from glaciers of southeastern Tibetan Plateau (1.96±0.66 Gg yr^–1^) is a little lower than that by Liu et al. [31] (~3 Gg yr^–1^).

**Fig 9:**
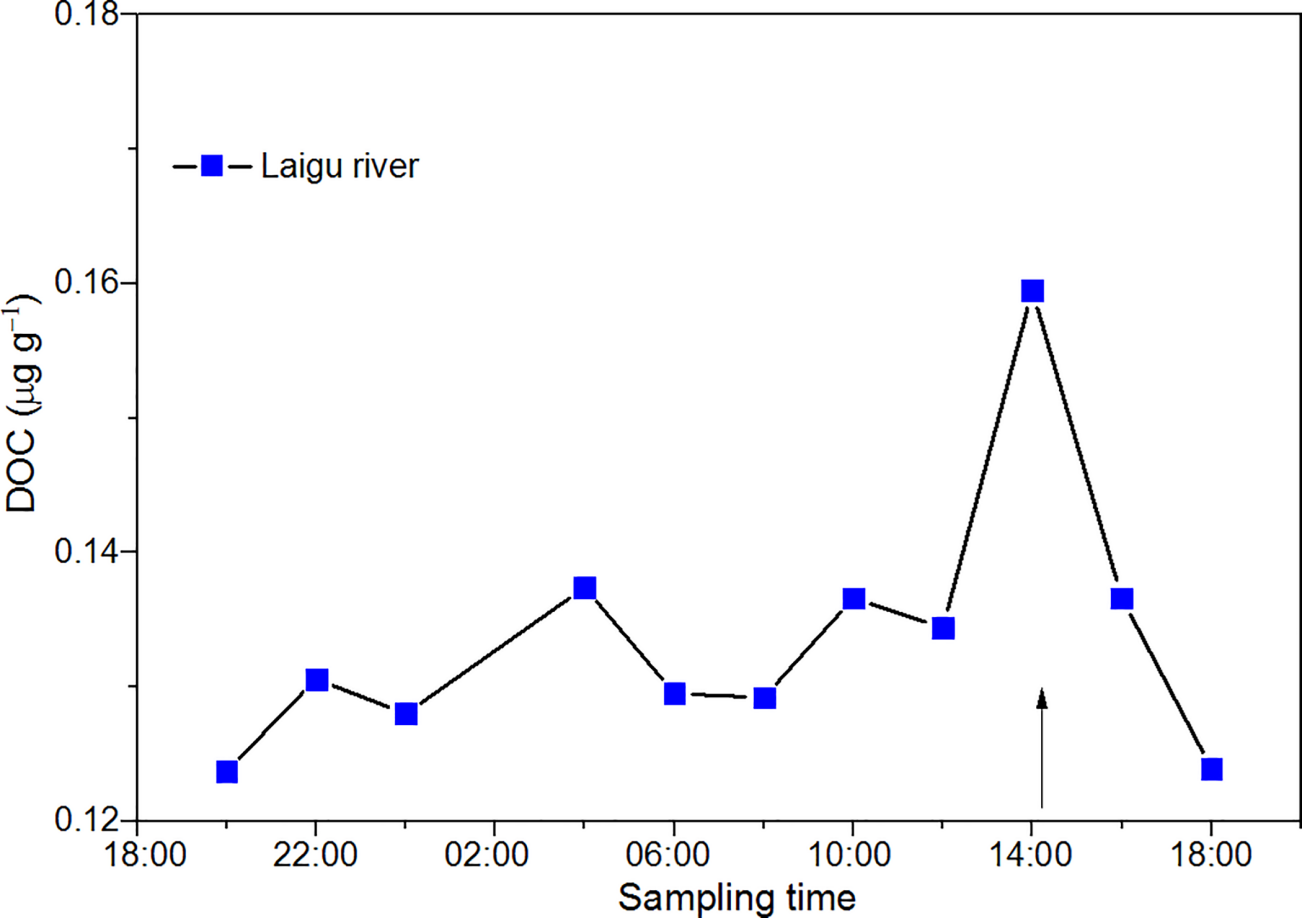
Diurnal variations of DOC concentration in Laigu glacier river samples.

DOC export from glaciers can also affect DOC concentration in river water. The diurnal variations of DOC concentrations from the Laigu glacial river water samples showed higher values after 14:00 (Fig 9). Similarly, Sun et al. [64] also found that mercury in the glacier-fed river water exhibited significant diurnal variations with greater concentrations during high flow periods in the afternoon. Han et al. [65] and Singh et al. [66] noted that diurnal variations in glacial runoff were pronounced as a result of glacier melt. The consistency of DOC with that of the runoff suggests that glacier ablation intensity has a strong influence on the DOC concentrations in downstream river water. Hood et al. [19], Huntington et al. [67], and Lawson et al. [22] found that coastal ecosystems are sensitive to alteration of both the quantity and lability of terrigenous DOC exported from glaciers and delivered by rivers. For example, earlier snowmelt and spring runoff led to changes in the timing of DOC exported to the Gulf of Maine [67]. In Alpine glaciers, Singer et al. [14] found DOC to be highly diverse and that a significant fraction of this material was bioavailable, suggesting that glacier-derived DOC contributed to downstream carbon cycling in glacier-fed streams. As a large fraction of OC, estimation of POC export from glaciers still remained poorly constrained. In the central and southern Tibetan Plateau, the export of POC can be calculated to be about 10.1−20.6 Gg yr^-1^ (S3 Table). The continuous export of DOC and POC from glaciers will affect downstream ecosystems [14]. The quantification of this impact in mountainous regions is of great interest for risk assessment and adaptation.

### Implications and perspectives

Recent studies in the Tibetan Plateau have shown that glacial meltwater is enriched in dissolved carbonaceous species, which are essential for microbial community and downstream carbon cycling [14, 21–22]. In the northern Tibetan Plateau (Laohugou No. 12 glacier), Yan et al. [33] indicated that 46.2% of DOC in snow is bioavailable and can be decomposed into CO_2_ within one month of its release. The chemical composition of dissolved organic matter in cryoconite in Tibetan glaciers shows almost one-third of the identified dissolved organic matter molecules have low C/N ratios (≤20), indicating high bioavailability [48]. In this study, average C/N ratios is at 6.1±4.9 with a range from 2.0 to 22.5, further implying glacial originated DOC has high bioavailability. Estimation of the impacts of DOC from glaciers on downstream biogeochemistry and its relevance for carbon cycling in glacier-fed stream is still of great scientific interest on the Tibetan Plateau.

Diagnostic dual-isotope signatures (Δ^14^C/δ^13^C) of carbonaceous aerosol can be used to determine contributions from different sources [68–69]. For example, carbon isotopic signatures combined with a three-source mixing model showed that DOC deposited in snow across the icefield of southeast Alaska reflects fossil fuel combustion products (43%−73%) and, to a lesser extent, marine (21%−41%) and terrestrial sources (1%−26%) [9]. BC compositions in Himalayan glaciers indicate equal contributions from fossil fuel (46%) and biomass (54%) combustion, whereas BC in the remote northern Tibetan Plateau is predominantly derived from fossil fuel combustion (66%) [68]. Potential sources of carbonaceous aerosol in the southeastern Tibetan Plateau are discussed qualitatively; the quantification of the contributions to DOC deposition in glaciers from different sources needs to be investigated further in this region.

## Conclusions

In this study, we find that DOC concentrations in glaciers of southeastern Tibetan Plateau are slightly lower than other results from the Tibetan Plateau, but comparable to those in the Alps and Alaska. We found snow pit POC to be higher than that from the central Tibetan Plateau, but comparable to those from the margin regions of the Plateau. Mass fraction of organic and inorganic components in snow implied that carbonaceous components dominated the impurities in snow, characterized by contribution to more than 80% of the total mass. We found no systematic variations for DOC and TN with snow pit depth in the studied glaciers, but POC and BC showed consistent variations. High values of DOC occurred in the ice layer or dusty layers, similar to the pattern observed from Laohugou No.12 glacier in the northern Tibetan Plateau.

We estimated DOC export from glaciers to be 1.88–2.12 Gg yr^–1^, which was much lower than that from previous studies because of the different DOC concentrations used. We estimated export of POC from the study area to be 4.47–8.34 Gg yr^–1^, indicating that OC in glaciers of southeastern Tibetan Plateau may play an important role on the carbon cycling in downstream as a result of the accelerated glacier melt occurred in this region.

Using backward trajectories and CALIPSO images, we found that biomass burning from South Asia is also a major source of DOC deposition on glaciers of the Tibetan Plateau. We found that, as a result of the different loadings of DOC and other components, biological processes in southeastern Tibetan glaciers may play a crucial role on DOC characteristics as shown by other studies. This issue needs further study.

Our findings suggest that anthropogenic aerosols contribute abundant DOC to glaciers in the southeastern Tibetan Plateau. Consequently, the pronounced rate of glacial melting in this region may be delivering increased quantities of relic DOC to downstream of rivers. As global warming, climate change motivated glacier runoff can affect hydrological processes. Constrain the role of glacier/ice sheet in OC storage, deposition and export, and its link with terrestrial carbon fluxes will enhance our understanding of global carbon cycle.

## Supporting information

S1 Table Detailed information of sampling sites from Yarlong, Dongga, Renlongba, and Demula glacier in the southeastern Tibetan Plateau. (SP represents snowpit).
(DOCX)

S2 Table Principle component analysis (PCA) for DOC and other proxies in snow of glaciers in the southeast Tibetan Plateau. (DOCX)

S3 Table Estimation of export DOC and POC from glaciers in the Tibetan Plateau. (DOCX)

S1 Dataset csv including sampling information, DOC data used in this study, and major ions & elements concentrations used in this study. (CSV)
