# Supplementary material for: Dissolved organic carbon in glaciers of the southeastern Tibetan Plateau: Insights into concentrations and possible sources
Source: PLoS One. 2018 Oct 11;13(10):e0205414. doi: 10.1371/journal.pone.0205414 (PMC6181362; doi:10.1371/journal.pone.0205414)
Supplement: S1 Table — (SP represents snowpit). (DOCX) [file pone.0205414.s001.docx]

**S1 Table Detailed information of sampling sites from Yarlong, Dongga, Renlongba, and Demula glacier in the southeastern Tibetan Plateau. (SP represents snowpit)**

| **Yarlong Glacier (YL)** | | | | | |
| --- | --- | --- | --- | --- | --- |
| Sampling date | Code | Snow/ice type | Latitude (N) | Longitude (E) | Elevation (m) |
| 2015.06.10 17:00 | YL1 | Aged snow | 29°18ʹ26.49ʺ | 96°46ʹ24.92ʺ | 4030 |
| 2015.06.10 17:20 | YL2 | Aged snow | 29°18ʹ24.94ʺ | 96°46ʹ24.92ʺ | 4054 |
| 2015.06.10 17:40 | YL3 | Aged snow | 29°18ʹ20.23ʺ | 96°46ʹ26.88ʺ | 4052 |
| 2015.06.10 17:55 | YL4 | Aged snow | 29°18ʹ17.05ʺ | 96°46ʹ26.07ʺ | 4041 |
| 2015.06.10 18:10 | YL5 | Aged snow | 29°17ʹ09.22ʺ | 96°48ʹ05.11ʺ | 4124 |
| **Dongga glacier (DG)** | | | | | |
| Sampling date | Code | Snow type | Latitude | Longitude | Elevation (m) |
| 2015.06.15 14:09 | DG-SP | Snow pit | 29°13ʹ34.97ʺ | 96°52ʹ30.53ʺ | 4705 |
| 2015.06.15 14:50 | DG-1 | Aged snow | 29°13ʹ39.38ʺ | 96°52ʹ28.63ʺ | 4650 |
| 2015.06.15 15:57 | DG-2 | Aged snow | 29°13ʹ47.24ʺ | 96°52ʹ32.91ʺ | 4570 |
| 2015.06.15 16:10 | DG-3 | Bare ice | 29°13ʹ59.08ʺ | 96°52ʹ28.92ʺ | 4490 |
| 2015.06.15 16:25 | DG-4 | Bare ice | 29°13ʹ34.97ʺ | 96°52ʹ30.53ʺ | 4705 |
| 2015.06.15 16:40 | DG-5 | Bare ice | 29°13ʹ39.38ʺ | 96°52ʹ28.63ʺ | 4650 |
| 2015.06.15 16:55 | DG-6 | Bare ice | 29°13ʹ47.24ʺ | 96°52ʹ32.91ʺ | 4570 |
| **Renlongba Glacier (RLB)** | | | | | |
| Sampling date | Code | Snow type | Latitude | Longitude | Elevation (m) |
| 2015.06.16 14:07 | RLB-SP | Snowpit | 29°14ʹ33.51ʺ | 96°55ʹ29.67ʺ | 4854 |
| 2015.06.16 14:20 | RLB-1 | Aged snow | 29°14ʹ35.55ʺ | 96°55ʹ31.86ʺ | 4847 |
| 2015.06.16 14:30 | RLB-2 | Aged snow | 29°14ʹ42.60ʺ | 96°55ʹ37.36ʺ | 4818 |
| **Demula Glacier (DML)** | | | | | |
| Sampling date | Code | Snow type | Latitude | Longitude | Elevation (m) |
| 2015.06.12 14:33 | DML-SP | Snowpit | 29°21ʹ09.33ʺ | 97°01ʹ14.22ʺ | 5138 |
| 2015.06.12 16:00 | DML-1 | Fresh snow | 29°21ʹ05.74ʺ | 97°01ʹ16.06ʺ | 5111 |
| 2015.06.12 16:25 | DML-2 | Fresh snow | 29°21ʹ04.23ʺ | 97°01ʹ17.45ʺ | 5190 |
| 2015.06.12 16:45 | DML-3 | Fresh snow | 29°20ʹ54.85ʺ | 97°01ʹ19.20ʺ | 5050 |
| 2015.06.12 17:10 | DML-4 | Fresh snow | 29°20ʹ47.17ʺ | 97°01ʹ24.07ʺ | 5002 |
| 2015.06.12 16:00 | DML-5 | Bare ice | 29°21ʹ06.85ʺ | 97°01ʹ14.04ʺ | 5117 |
