# Supplementary material for: Dissolved organic carbon in glaciers of the southeastern Tibetan Plateau: Insights into concentrations and possible sources
Source: PLoS One. 2018 Oct 11;13(10):e0205414. doi: 10.1371/journal.pone.0205414 (PMC6181362; doi:10.1371/journal.pone.0205414)
Supplement: S2 Table — (DOCX) [file pone.0205414.s002.docx]

**S2 Table Principle component analysis (PCA) for DOC and other proxies in snow of glaciers in the southeast Tibetan Plateau.**

|  | Vector Component | | | | Extraction Component (%) | | | |
| --- | --- | --- | --- | --- | --- | --- | --- | --- |
|  | PCA1 | PCA2 | PCA3 | PCA4 | PCA1 | PCA2 | PCA3 | PCA4 |
| DOC | -0.261 | 0.196 | 0.150 | 0.790 | 6.79 | 3.86 | 2.25 | **62.41** |
| POC | 0.973 | -0.026 | -0.037 | -0.040 | **94.75** | 0.07 | 0.14 | 0.16 |
| EC | 0.952 | 0.020 | 0.054 | 0.267 | **90.65** | 0.04 | 0.30 | 7.11 |
| TN | 0.049 | 0.273 | 0.898 | 0.225 | 0.24 | 7.45 | **80.73** | 5.08 |
| Cl^−^ | 0.540 | -0.078 | 0.226 | 0.706 | **29.14** | 0.61 | 5.12 | **49.85** |
| SO_4_^2−^ | 0.017 | 0.893 | 0.424 | 0.032 | 0.03 | **79.73** | 18.01 | 0.10 |
| NO_3_^−^ | -0.079 | 0.466 | 0.847 | 0.111 | 0.63 | 21.68 | **71.74** | 1.23 |
| NH_4_^+^ | 0.056 | 0.271 | 0.922 | 0.173 | 0.31 | 7.32 | **85.03** | 3.01 |
| Na^+^ | 0.937 | 0.147 | 0.014 | -0.141 | **87.76** | 2.18 | 0.02 | 1.99 |
| Mg^2+^ | 0.105 | 0.946 | 0.250 | 0.148 | 1.10 | **89.42** | 6.27 | 2.20 |
| Ca^2+^ | 0.030 | 0.946 | 0.260 | 0.103 | 0.09 | **89.56** | 6.74 | 1.07 |
| K^+^ | 0.457 | 0.262 | 0.495 | 0.626 | **20.89** | 6.88 | **24.51** | **39.14** |
| % of Variance | 46.58% | 26.73% | 12.34% | 7.30% |  |  |  |  |
| Cumulative % | 46.58% | 73.31% | 85.65% | 92.95% |  |  |  |  |
