# Supplementary material for: Dissolved organic carbon in glaciers of the southeastern Tibetan Plateau: Insights into concentrations and possible sources
Source: PLoS One. 2018 Oct 11;13(10):e0205414. doi: 10.1371/journal.pone.0205414 (PMC6181362; doi:10.1371/journal.pone.0205414)
Supplement: S3 Table — (DOCX) [file pone.0205414.s003.docx]

**S3 Table Estimation of export DOC and POC from glaciers in the Tibetan Plateau**

| Glacier regions | Southeastern TP | Mount Altai  Mushitaoling  Tienshan | Mount Karakoram  Pamirs  Mount Kunlun  Mount Altunshan | Mount Tanggula  Qiangtang Plateau  Kailas Tange | Himalayas  Mount Nyainqntanglha  Mount Hengduan | Total |
| --- | --- | --- | --- | --- | --- | --- |
| Glacier volume (km3) |  | 718.85 | 1975.93 | 887.41 | 911.8 | 4493.99 |
| Glacier area (km2) | 10699 | 7367.52 | 21565.27 | 11878.96 | 10954.26 | 51766.01 |
| Average DOC conc.  (μg g^-1^) | 0.16 | 0.76 | 1.10 | 0.91 | 1.26 |  |
| Average POC conc.  (μg g^-1^) | 0.38 | 1.74 |  | 0.61 | 1.38 |  |
| Amount of DOC storage (Tg) |  | 0.46 | 1.84 | 0.68 | 0.98 | 3.96 |
| Amount of POC storage (Tg) |  | 1.25 |  | 0.54 | 1.26 |  |
| Glacier mass balance (mm w.e. yr^-1^) | -1100 | -133 | +250 | -550 | -400 to -1100 |  |
| DOC export (Gg yr^-1^) | 1.96±0.66 | 0.74 |  | 5.95 | 5.52~15.18 |  |
| POC export (Gg yr^-1^) | 5.88±2.15 | 2.13 |  | 3.98 | 6.05~16.6 |  |

Note:

1. Glacier volume and area cited from [Guo et al., 2015]
2. Glacier mass balance cited from [Yao et al., 2012]
3. Tienshan glacier mass balance [Xu et al., 2018]
4. Average DOC and POC concentrations can be checked in the [Liu et al., 2016], and Table 1 and Table 2 in the main text.

**References:**

Guo W, Liu S, Xu J, Wu L, Shangguan D, Yao X, and others. The second Chinese glacier inventory: data, methods and results. J Glaciol. 2015; 61(226). https://doi.org/10.3189/2015JoG14J209

Liu Y, Xu J, Kang S, Li X, Li Y. Storage of dissolved organic carbon in Chinese glaciers. J Glaciol. 2016; 62 (232), 402−406. https://doi.org/10.1017/jog.2016.47

Xu M, Wu H, Kang S. Impacts of climate change on the discharge and glacier mass balnce of the different glacierized watersheds in the Tianshan Mountains, Central Asia. Hydrol Process. 2018; 32, 126-145. <https://doi.org/10.1002/hyp.11409>

Yao T, Thompson LG, Yang W, Yu W, Gao Y, Guo X, et al. Different glacier status with atmospheric circulations in Tibetan Plateau and surroundings. Nat Clim Change. 2012a; 2 (9), 663–667. https://doi.org/10.1038/nclimate1580
